# Renegotiating inter-professional boundaries in maternity care: implementing a clinical pathway for normal labour

**DOI:** 10.1111/1467-9566.12096

**Published:** 2014-03-19

**Authors:** Billie Hunter, Jeremy Segrott

**Affiliations:** 1 School of Health Care Sciences, Cardiff UniversityUK; 2 Cardiff School of Social Sciences, Cardiff UniversityUK

**Keywords:** boundary work, inter-professional relationships, professional project, clinical pathways, normal labour, midwifery, obstetrics

## Abstract

This article presents findings from a study of a clinical pathway for normal labour (Normal Labour Pathway) implemented in Wales, UK. The study was conducted between 2004 and 2006. The pathway aimed to support normal childbirth and reduce unnecessary childbirth interventions by promoting midwife-led care. This article focuses on how the pathway influenced the inter-professional relationships and boundaries between midwives and doctors. Data are drawn from semi-participant observation, focus groups and semi-structured interviews with 41 midwives, and semi-structured interviews with five midwifery managers and six doctors, working in two research sites. Whereas some studies have shown how clinical pathways may act as ‘boundary objects’, dissolving professional boundaries, promoting interdisciplinary care and de-differentiating professional identities, the ‘normal labour pathway’ was employed by midwives as an object of demarcation, which legitimised a midwifery model of care, clarified professional boundaries and accentuated differences in professional identities and approaches to childbirth. The pathway represented key characteristics of a professional project: achieving occupational autonomy and closure. Stricter delineation of the boundary between midwifery and obstetric work increased the confidence and professional visibility of midwives but left doctors feeling excluded and undervalued, and paradoxically reduced the scope of midwifery practice through redefining what counted as normal.

In the sociology of health and medicine there has been a longstanding interest in processes of professionalisation and the social construction of professional identities. Attention has particularly focused on how ‘professional projects’ claim ownership of bodies of knowledge and exercise control over the work informed by this knowledge, affording protection from competing professions (Powell and Davies 2012). A central part of this ‘identity work’ is the formation and maintenance of intra-professional and inter-professional boundaries, often achieved through the employment of legitimising discourses (Sanders and Harrison 2008), and boundary work that draws on competing ideologies and the creation of ‘contrasting cases’ (Gieryn 1983). Professions may seek to legitimise their distinctive identity by highlighting its scientific basis, its treatment philosophy, its uniqueness (or similarities with other contrasting professions) or how it contributes to the efficient organisation of care (Sanders and Harrison 2008). As Gieryn (1983) argued, boundary work is most common when there is a desire to maintain occupational autonomy against threats from competitors, to expand an occupational sphere or to monopolise a domain. While Gieryn′s account of boundary work was concerned with the early development (and demarcation) of science as a discipline, he argues that these processes are generalisable to the study of other professions (such as medicine), since ‘expansion, monopolization and protection are generic features of “professionalization” ’ (p. 792).

The salience and complexity of professional boundary work has been accentuated by recent healthcare system developments emphasising inter-professional collaboration and workforce flexibility. These developments are often embedded within new soft technologies, including protocols and clinical pathways aimed at levering evidence into practice, standardising practice in line with this evidence and reconfiguring who delivers care and how. These technologies bring together a range of professional interests (Allen 2009), potentially altering professional identities and boundaries (Pinder *et al*. 2005). In particular, they can enable ‘lower status’ occupational groups, such as nurses and midwives, to expand their professional base, thus challenging the traditional dominance of the medical profession (Powell and Davies 2012). Nancarrow and Borthwick (2005: 901) capture this neatly, suggesting that ‘For established or aspiring professions, occupational strategies often centre on the protection and maintenance of boundaries, coupled with an ongoing campaign to expand areas of control.’ Expansion of professional territory typically focuses on incorporating higher status roles, ‘whilst delegating lower status roles to subordinate groups’ such as healthcare assistants (Nancarrow and Borthwick 2005: 902). Thus the emergence of new professional identities through processes of role specialisation, diversification and substitution (both horizontal and vertical) (Nancarrow and Borthwick 2005, Sanders and Harrison 2008) is a key feature of the contemporary healthcare system, with a number of professional projects jostling for occupational jurisdiction. These professional projects are typified by their attempts to assert their profession′s specialist skill base and unique expertise. However, as McDonald *et al*. (2009) suggest, there is an important distinction to be made between exclusionary strategies (concerned with intra-occupational control and the eligibility to practice) and demarcation strategies, which attempt to define and control the boundaries between different professions.

At the shifting boundaries of medicine and the midwifery and nursing professions a complex set of identity work is therefore taking place. As well as taking on roles that doctors have readily relinquished (Charles-Jones *et al*. 2003), midwives and nurses may also be seeking to expand (or re-establish) their professional territory in ways which the medical profession resists (Powell and Davies, 2012). As Larson (1990: 45) argues, ‘professionals and experts, whenever they are challenged, individually or collectively, retrench behind the boundaries of their discursive fields and retreat towards the protected core’. Drawing on Larson′s work, Martin *et al*. (2009:1192) highlight how this ‘retreat to the core’ may operate in the case of dominant professions such as medicine:

[E]stablished professions tend to cede their core work only reluctantly, using an armoury of techniques to defend their territory. By making claims to scientific or specialist expertise, for example, dominant professions are able to set the terms of reference of such territorial battles, so that challengers are immediately weakened by the need to appeal to the discursive norms of the dominant professions.

While the notion of boundaries might suggest clear (if contested) divisions between professions (akin to tectonic plates?), there is evidence that as practitioners’ roles shift, boundaries may become less clearly demarcated (Carmel 2006). Such situations require significant boundary work by those involved, both through active negotiation and other ways of accomplishing social order (Allen 1997). Where professional roles overlap or different professionals are viewed as being equally skilled in performing particular tasks, tensions and uncertainties can arise, particularly if informal organisational systems are out of alignment with formal professional roles and boundaries (Carmel 2006).

## Clinical pathways

Clinical pathways are key examples of healthcare governance initiatives aimed at improving the quality of care by reducing undesirable variations in practice and standardising care delivery (Allen 2009). Usually designed by local-level multidisciplinary teams to tackle local problems, they represent ‘ideal’ patient journeys that map out healthcare activities and specify the input of various professionals (Atwal and Caldwell 2002, Currie and Harvey 2000). As Gittell and Weiss (2004) argue, clinical pathways specify what care should be provided for patients with a particular condition, who should provide it, and how its constituent tasks should be sequenced. A growing body of sociological critique has explored the ways in which pathways may reshape practice and influence inter-professional identities and relationships (Allen 2009, Pinder *et al*. 2005). Allen (2009: 355) proposes that clinical pathways may function as boundary objects: ‘an object which inhabits several social worlds and fulfils a role in structuring relations between them’. Exploring why some boundary objects are more effective than others in mediating between different communities of practice, Fox (2011) suggests that shared social meaning is all important.

As boundary objects, pathways are frequently aimed at encouraging dialogue across professional boundaries, often functioning as a shared language for discussing patient care (Allen 2009, Cabitza *et al*. 2008). Indeed, it is often in establishing inter-professional communication (rather than standardising care itself) that they are most effective (Atwal and Caldwell 2002, Currie and Harvey 2000, Hunter and Segrott 2008). Accounts of successful initiatives often refer to practitioner consensus: that patient care can be best achieved by incorporating multiple professional views (Caminiti *et al*. 2005). So, from Fox′s (2011) perspective, these pathways function as facilitative boundary objects and are thus more likely to become implemented in practice.

Drawing on Berg′s (1997) analysis of protocols (close relations of clinical pathways), we have previously argued that pathways are not ideologically neutral but are written by specific authors incorporating ‘particular kinds of information and rationalities, raising questions of exclusion, power and governance’ (Hunter and Segrott 2008: 609). Thus, clinical pathways may be thought of as ‘regulatory mechanisms’ designed to ‘control, monitor and manage the process of healthcare’ (Barnes, 2000: 201). Moreover, they do not operate within a vacuum and must be understood within their broader social and ideological context. For example, convening differing professionals to map patient journeys inevitably exposes competing inter-professional discourses and professional hierarchies. This can provide opportunities to break down inter-professional boundaries (Atwal and Caldwell 2002, Currie and Harvey 2000); however, it may also emphasise strongly held and conflicting perspectives (Barnes 2000, Pinder *et al*. 2005). When pathway formation requires the renegotiation of role boundaries, competing approaches representing fundamentally different ideologies of care are most clearly foregrounded (Berg 1997, Pinder *et al*. 2005).

## Midwifery and medicine

While professional identities and boundary work in health care have been extensively studied, limited attention has been given to how these processes play out in maternity care – a ‘highly charged mix of medical science, cultural ideas and structural forces’ (De Vries 2004: 15). Characterised by conflicting approaches to childbirth and competing claims for occupational jurisdiction (Abbott 1988), maternity care provides a fertile ground for sociological study, yet to date relatively few researchers have examined at a micro-level how the contested boundaries and claims for knowledge advanced by doctors and midwives are played out.

Analyses of 19^th^ and 20^th^-century struggles for occupational jurisdiction (Witz 1992) provide insights into the historical underpinnings of current day tensions between midwives and doctors. Historically, midwives have been the subordinate group within maternity care, working covertly behind the scenes to give ‘illusions of compliance’ (Hunter 2005: 260), rather than openly challenging biomedicine. These covert strategies form part of the ‘negotiated order’ (Strauss *et al*. 1985) of maternity care, the process by which social order is continuously reconstituted.

Boundaries are frequently drawn with reference to ‘normal birth’ (the realm of the midwife) and ‘abnormality’ (the obstetricians’ domain), although these culturally constructed categories are ambiguous and shifting, with fuzzy demarcation lines and a large grey area at their interface. Both professions are engaged in boundary work, drawing on various discourses to legitimise their expertise and authority and thus demonstrate their distinctive characteristics (Gieryn 1983, Sanders and Harrison 2008).

In common with other professions complementary or alternative to medicine, midwives legitimise their work by stressing their expertise and unique philosophy, employing a person-centred, ‘with woman’ discourse that emphasises holism, normal physiology and emotional support (Norris 2001, Sanders and Harrison 2008). Midwives contrast their philosophy with bio-medicine, which has been the dominant discourse for the past hundred years and is underpinned by claims to greater scientific knowledge and technical skill than those held by midwives. The midwifery discourse however frequently positions doctors as employing a reductive, interventionist approach that anticipates danger and emphasises risk, although this rather simplistic dichotomy fails to acknowledge a continuum of practice along which individual midwives and doctors are situated (Bryers and van Teijlingen 2010). Given the coexistence of differing approaches to childbirth and competing claims for authoritative knowledge, there have been surprisingly few sociological studies that focus specifically on inter-professional relationships in maternity care. This is even more remarkable, given that various reports have identified polarised professional positions and turf wars as key factors contributing to unsafe practice. For example, the Confidential Enquiry into Maternal and Child Health – ‘Why Mothers Die’ (Lewis 2005: 56) found a ‘lack of communication and teamwork’ contributed to a number of maternal deaths. This lack of research attention was noted over 20 years ago by Kitzinger *et al*. (1990). Exploring what they tellingly characterise as ‘traditionally antagonistic’ doctor–midwife relationships, they observe ‘conflicting views about each other′s legitimate spheres of concern’ (Kitzinger *et al*. 1990: 151), with inter-professional relationships ‘constantly under informal negotiation’ (p. 160). Recent social scientific studies confirm that these ideological and professional differences persist (McIntyre *et al*. 2012, Pollard 2011, Reiger and Lane 2009). For example, an organisational case study found Australian midwives and doctors had significantly different attitudes to childbirth and differing professional cultures. Both tended to negatively stereotype the other (Reiger and Lane, 2009), resulting in a climate of incivility and mistrust. Applying complexity theory to maternity care Downe *et al*. (2010) suggest that a self-perpetuating pattern may be established. Each professional group anticipates disharmony, reinforced by existing prejudices. Thus inter-professional tensions become ‘viral’ and are echoed at all organisational levels, presenting major challenges not only for safe practice but also for healthcare reforms requiring collaborative working.

Current UK maternity care policies are likely to affect the division of labour and exacerbate inter-professional tensions. Underpinned by professional and government concerns about increasing childbirth intervention, and supported by evidence of improved clinical outcomes for women and babies receiving midwife-led care (Hatem *et al*. 2008), a number of policies have been devised at local, regional and national levels aimed at encouraging normal birth and minimising unnecessary intervention (Department of Health 1993, 2007, Department of Health Social Services and Public Safety 2012, National Health Service Quality Improvement Scotland 2009, Welsh Assembly Government 2002). Although varying between the four UK countries, policies commonly identify midwives as the key professionals in straightforward childbirth. Welsh policy utilises a clinical pathway (the Normal Labour Pathway) to achieve these aims.

In these ways, midwifery is therefore seeking to expand its professional territory, renegotiating its boundaries and relationship with the medical profession. Midwives’ identity work may, however, prove particularly complex: although they may legitimise their professional identity by stressing its distinctive philosophy and differentiating it (in oppositional terms) from mainstream medicine, Foley and Faircloth (2003) found that midwives in the USA also utilised medicine as a ‘discursive resource’, sometimes stressing the scientific basis of midwifery or by emphasising their equality with doctors.

## Article overview

In this article we explore how the introduction of a maternity care clinical pathway in Wales, UK (the All Wales Clinical Pathway for Normal Labour, or ‘normal labour pathway′as it was known), led to the renegotiation of inter-professional relationships and boundaries between midwives and doctors. The research study was conducted in South Wales over a 2-year period. The aim was to explore how the pathway was used in real life settings and evaluate its implementation from the perspectives of all key players: midwives, doctors, mothers and midwifery managers. A policy ethnography approach provided insights into the pathway creation and implementation, as well as how it was used on the ground. We therefore aim to address Martin *et al*. (2009)′s concern that:

[T]he literature on the health professions concentrates on potential, rather than actual, shifts in professional boundaries: it considers legitimacy claims in isolation, rather than in relation to specific challenges to the professional division of labour. (2009: 1191)

The article fills a significant gap in the recent body of knowledge on midwife–doctor relationships by analysing the identity work undertaken to maintain inter-professional boundaries. It also provides novel insights into the use of a clinical pathway as an object of demarcation used to support and legitimise the professionalising strategies of midwives, rather than as a boundary object as more commonly described in the literature (Allen 2009).

## Description of study methods and approach

This study used a policy ethnography approach. Policy ethnography explores how policy is put into action from the viewpoint of the key players, thus increasing our understanding of organisations in action (Griffiths 2003). This approach facilitates a detailed consideration of processes, as it allows the investigation of a policy′s journey from initial conception to implementation. In particular, it enables us to investigate how polices are enacted on the ground, underpinned by the assumption that policies are made not only at the top by policymakers but are also constructed locally by grassroots workers to fit the realities of practice (Dubois 2009, Griffiths 2003). Ethnographic approaches are particularly appropriate for exploring organisational cultures and cultural change, as they have the potential to enhance understanding of the complex nature of knowledge translation, in particular why some policies are more easily implemented and accepted than others (Gabbay and Le May 2010).

Data were collected between October 2004 and October 2006 in two research sites, selected for their contrasting features; they differed in size, location, model of service delivery and timing of the ‘normal labour pathway’ implementation. Maternity Unit A was a medium-sized obstetric unit, based in a semi-rural area, with an annual birth rate of approximately 1400 births. The midwives worked mainly in community-based teams, providing midwife-led, continuity of care for women with straightforward pregnancies. This unit had implemented the pathway early in the national process. In contrast, Maternity Unit B was a large urban tertiary referral obstetric unit with an annual birth rate of approximately 3600 births. Service delivery was more traditional: the midwives worked in either hospital or community locations and midwife-led continuity of care was rare. This unit implemented the pathway late in the national process.

Data were collected in three phases. In phase one, semi-structured interviews were conducted with senior level midwives and doctors involved in the initial pathway development. To protect participant anonymity, these are referred to as key informants and no further identifying details are provided. Analysis of a range of relevant documents was also undertaken, but is not referred to in this article. Phases two and three were conducted in maternity units A and B, respectively. The process began with semi-participant observation of six midwives using the pathway while they cared for women in normal labour, followed by an unstructured interview. A total of 30 hours of observational fieldwork was undertaken by the principal investigator. There was the potential that the woman′s clinical condition and risk status could change during the observation, requiring her to ‘exit the pathway’. In this eventuality, it was planned that the observation would be stopped but the observational field notes up to that point would be included in the data, in order to provide deeper insights into the use of the pathway and how it might contribute to the decision-making process. However, as it turned out, none of the observed labours resulted in women exiting the normal labour pathway.

Insights gained from the initial data collection were used to develop focus group and semi-structured interview schedules for use with midwives, managers and doctors. All participants were encouraged to discuss their experiences of pathway development and implementation, and how the pathway had affected their practice. Focus group discussions were effective in enabling insights into shared experiences, in particular the pathway′s effect on midwifery culture and midwives’ working practices. All focus groups and interviews were conducted by the principal investigator and a research assistant.

Study approval was obtained from the local research ethics committees and research and development committees linked to the participating sites. Following permission to access the sites from gatekeepers (obstetric and midwifery leads), briefings were given at staff meetings and information letters were sent to all maternity staff inviting their participation. The participants were required to have current or past experience of the pathway. Potential participants contacted the research team and the midwives were asked to select their preferred mode of participation (observation plus interview or focus group). There was considerable interest in the study and recruitment of participants did not present any difficulties. It is acknowledged that this purposive sampling strategy had the potential to recruit participants with strongly held views about the pathway, positive or negative.

A total of 56 participants took part (see Table1): four senior key informants involved in initial pathway development and implementation and 41 midwives, six doctors and five midwifery managers from the two maternity units. A sample of women who were cared for on the pathway was also interviewed; their responses will be discussed in a forthcoming article. In all, 41 midwives participated, 21 from Unit A, and 20 from Unit B. Their length of clinical experience varied between one year and 35 years, with those from Unit A having longer clinical experience than those from Unit B (see Table2). Doctors and managers were drawn from both sites and represented varying lengths of experience. Three doctors participated per site and represented different clinical grades (at registrar and above). Given the small sample size, no further details are given for doctor and manager participants in order to protect anonymity.

**Table 1:** Details of sample and data collection methods

Data collection method	Participant type	Sample size
Interviews	Key informant	4
Observations and interviews	Midwives	6
	Mothers	5
Focus groups (7)	Midwives	31
Semi-structured interviews	Midwives	4
Semi-structured interviews	Doctors	6
Semi-structured interviews	Midwifery managers	5
Semi-structured Interviews	Mothers	10
Interviews	Key informant	4

**Table 2:** Details of midwife participants – length of clinical midwifery experience

Length of experience	Unit A	Unit B
1–5 years	3	10
6–10 years	2	4
11–35 years	16	6

### Data analysis

Detailed field notes were kept during the observations and written up in full immediately afterwards. Interviews and focus group discussions were audio-recorded and later transcribed and entered into the computer assisted qualitative analysis package N6. All data and field notes were analysed thematically by the principal investigator and research assistant. Both researchers read the transcripts and field notes several times, identifying minor and major codes and the relationships between them (Coffey and Atkinson 1996). In order to enhance analytic trustworthiness and rigour this process was undertaken blind, as a form of peer validation. After minor refining of coding categories and terminology, researcher agreement was reached. An analytic coding frame was created and used to code all data. At the end of the study, a project roadshow travelled around Wales to allow early dissemination of the findings to midwives and doctors. An additional purpose of the Roadshow was to test out the analysis during audience discussion and via written evaluation forms. This feedback suggested that the findings concurred with individuals’ experiences. While they are not a formal means of respondent validation, the roadshows do indicate the general credibility of the data.

## Findings

This article focuses on selected findings relating to the effect of the pathway on inter-professional relationships and boundaries. It draws largely on the interview and focus group data, interwoven with fieldwork observations when relevant. Interpretation of the data is informed by sociological understandings of boundary work and how clinical pathways may act at the interface of professional boundaries, signalling the limits of delegated authority. Key themes identified are:

- Legitimising a midwifery model of care
- Redefining midwifery territory
- Shifting medical territory and jurisdiction
- Redefining the doctor′s role

These selected findings are situated within the broader analytic framework (Hunter 2007), which included: pathway creation and implementation (Hunter 2010); effects on midwives’ work (Hunter and Segrott 2010); effects on midwives’ relationships with doctors and mothers, and the macro-level effects on Welsh maternity care (Hunter and Segrott 2010).

The ‘normal labour pathway’ is a three-part document used only by midwives while providing midwife-led intrapartum care for low-risk women in normal labour. The pathway uses the definitions of normal labour and risk status established by the National Institute for Health and Clinical Excellence (NICE); originally those set out in 2001 (NICE, 2001), and updated in 2007 in line with its guidelines for intrapartum care (National Collaborating Centre for Women and Children′s Health 2007). The pathway′s aim is to support midwives in promoting normal childbirth by using low-tech strategies, specified in an algorithm, that enhance normal birth physiology, such as encouraging maternal movement and upright position (Hunter and Segrott 2010). The first example of a pathway aimed at promoting normal birth, its ultimate purpose is to reduce unnecessary childbirth intervention (Fox 2004). The pathway generated substantial interest and subsequently other clinical pathways have proliferated in maternity care, including variations of the Welsh pathway (Rycroft-Malone *et al*. 2008). However, there has been no evaluation of the clinical advantages or disadvantages of the ‘normal labour pathway’ for mothers and babies, and thus there is no evidence to support the claims made for its efficacy and value (Hunter and Segrott 2010). The pathway is accessible electronically.

The ‘normal labour pathway’ shares some similarities with classic clinical pathways. For example, it contains a decision-making protocol, informed by research evidence, where available, and best practice where no research base was identified. The pathway also acts as a record of care, in the form of a tick box with written notes made only if there is non-compliance with usual care (Hunter and Segrott 2008). However, the ‘normal labour pathway’ has unique and distinctive features. Firstly, it is unidisciplinary – used only by midwives. This removes the challenges – and possible opportunities – of creating and using a multidisciplinary tool although, as will be seen, other challenges have arisen instead. Secondly, rather than standardising care for a specific condition, the pathway set out to fundamentally change the approach to intrapartum care by creating a tool to implement midwife-led care for all women with straightforward pregnancies. This was achieved by devising clear eligibility criteria demarcating professional boundaries: all women cared for on the pathway were midwife cases, with medical contact only if a deviation required obstetric intervention. Increased time was allowed for labour progress so women could remain in midwife-led care for longer. Finally, the pathway was a national rather than a local strategy, embedded in Welsh maternity policy (Welsh Assembly Government 2002).

The ‘normal labour pathway’ had intended and unintended consequences for inter-professional relationships, which were consistent across both research sites. An intended consequence was the sharper delineation of midwifery–obstetric boundaries and the increased visibility of midwife-led care. Midwives described the pathway as being supportive of a normal, physiological approach to intrapartum care, enhancing confidence in midwifery knowledge and skills and providing clear criteria for midwife-led care. Unsurprisingly, midwives did not generally describe this privileging of a midwifery discourse as problematic. In contrast, doctors expressed negative views about the pathway′s effect on their identity and scope of practice. They described feeling excluded and undervalued. These experiences and the increased inter-professional tensions that resulted were unintended. However, we will argue that conflicts were certainly predictable, given that from its conception the pathway was a professional project intended to promote midwife-led care, and facilitating inter-professional working was never the intention of the pathway developers.

## A midwifery project from the outset?

Although increasing levels of childbirth intervention could reasonably be seen as a problem for all maternity care practitioners, the approach taken in Wales was not multi-professional. A decision had apparently already been made by senior midwives and at a government level: the solution was to increase midwife-led care, using a clinical pathway to achieve this (Hunter 2010). Consequently, some midwife participants argued that it was not really necessary to engage doctors other than in a support role:

We needed a body of clinicians (for the steering group) and we discussed the balance of midwives versus obstetricians, and the internal reference group – the policymakers – and [senior civil servant] believed it was a midwifery initiative but that we would absolutely want the support of obstetricians. Because it can′t work in isolation. (Interview key informant)

The eventual steering group composition had a majority of midwives and only one obstetrician member. Other participants commented on this imbalance, and its implications:

It would have helped if they [doctors] were more involved in the implementation and the development … they should have had more than one on it – if you want to implement something new, you need to get lots of stakeholders on board. (Interview S, manager Unit B)

We were made aware of it but I felt we had no say in the matter. Right from the beginning we disagreed with some of the stuff in there. I do think if you want to get the doctors on board, you need to do more of an effort. (Interview J, doctor Unit B)

### Legitimising a midwifery model of care

Creating and implementing the ‘normal labour pathway’ emphasised differences in professional attitudes and approaches to childbirth, particularly during initial implementation, with negative effects on collaborative working. Midwives and doctors working in both research sites and across all care delivery settings commented that relationships had become ‘us and them’ since the pathway′s introduction, with doctors being alarmed by the midwives’ ‘territorial behaviour’ and the midwives justifying this as a necessary step in shifting the balance of professional power.

Many midwives from both units described how the pathway had given them ‘the OK’; in effect legitimising a midwifery approach that anticipated a normal birth and encouraged normal physiology. This sense of having had permission to challenge a medicalised approach to labour care led to a sense of increased confidence, especially for newly qualified midwives:

I think it′s helped me to have the confidence really to practice with the way I wanted to practice … it gives you a little bit more confidence to say ‘This isn′t what the normal pathway suggests and this woman falls into the normal pathway therefore this is what I am going to do’. (Focus group 4, Unit A: recently qualified midwife)

Experienced midwives also admitted that the pathway had boosted their confidence, particularly by giving them ‘permission’ in relation to the timing of progress in labour:

It has sort of given midwives permission not to time labour in the way it was timed previously … in some ways that′s the biggest thing it′s done for me – is that the timing of labour is less rigid than it was. (Interview J, midwife Unit B)

To legitimise their practice the midwives drew on both the normal birth discourse embodied by the pathway and the discourse of scientific evidence-based practice exemplified by the reference list included in the pathway, which underpinned its guidance:

Midwife 1: It′s backed by research, which is really how midwifery should be practised, rather than that′s how it′s always been done blah blah.
Midwife 2: It′s written down and because it′s coming from research, you′ve got all the references in front of you as to what type of research has been used and it sort of … just backs you up. (Focus group 5, Unit B midwives)

This scientific discourse was also described as offering a protective function in relation to interactions with the medics:

Midwife 1: A good thing is that you′ve got it all written out and everything is referenced. And really, if the woman is on the normal care pathway, there shouldn′t be any medics coming in saying ‘What is she doing? Why are you doing … ?’
Midwife 2: Yes, it′s protection for us in a way isn′t it? … I like using the pathway because it gives you a sense of greater autonomy. (Focus group 2, Unit A midwives)

Being an evidence-based initiative gave the pathway authority in the midwives’ eyes, which in turn legitimised a midwifery approach to care. However, it was apparent that the quality of the underpinning evidence was largely taken for granted by the midwives. By contrast, the doctors questioned the robustness of the evidence base:

We′re swapping one lot of vague-ish evidence for another lot of vague-ish evidence – and wait and see if anything goes wrong or not. (Interview J, doctor Unit B)

The pathway′s authority and legitimacy was further enhanced by its incorporation into Welsh policy:

Midwife 1: It makes it a bit more powerful … particularly if you are getting criticism from the doctors to be able to say ‘This is an All Wales – this is not just some little quirk that we thought up here’. (Focus group 1, Unit A midwives)

Midwives had previously tackled these issues as individuals rather than as a professional group. They had often experienced difficulties in defending a normal midwifery approach, tending to ‘do good by stealth’ (Kirkham 1999: 736); that is, they would use covert strategies such as giving ‘toast and a drink … sneakily’, as in the following account:

Now you think nothing of giving them a piece of toast and a drink when they are in the early stages of labour, whereas before you didn′t have permission to do it, you were doing it off your own back, rather sneakily, in case the people in charge saw you and didn′t like it. (Focus group 5 Unit B)

For some midwives the very existence of the pathway appeared to be enough to give a sense of back up. When asked directly, many midwife participants found it difficult to articulate exactly how the pathway achieved this, suggesting it had an almost talismanic quality:

Midwife: I think it′s [the pathway] really good for us as team midwives because we don′t intervene unless we really need to and we try and keep it as normal as possible anyway, but it does keep you more focused.
Facilitator: Is that because of the evidence that′s written down in there?
Midwife [doubtful tone]: I don′t think it′s as much the evidence – as much as it′s giving you the OK to do that … You don′t have to justify what you are doing because it is there. You know it′s there to back you up. (Interview N, midwife Unit A)

Throughout these data runs an image of midwifery as an embattled profession, with midwives seeking permission, protection and back up from the pathway to boost professional legitimacy and exclude those who seek to undermine it. This image is reinforced by midwives’ frequent use of battlefield metaphors:

It does give you a little bit of ammunition. (Focus group 2, Unit A midwives)

It′s ammunition against the doctors, which is an awful thing to say, but it has given you something, that you know from your evidence that you can support what you practice. (Interview N, midwife Unit A)

### Redefining midwifery territory

The ‘normal labour pathway’ clearly delineated midwifery territory in several ways. Perhaps most importantly, the pathway is a textual representation of midwifery knowledge accessed and used only by midwives. This exclusivity has important implications for establishing an authoritative knowledge base and the exercising of power.

In order to define professional territory the scope of the work, its recipients and boundaries must be specified. By identifying some women as appropriate for care solely by midwives the pathway clearly demarcates these territories. What is significant (and new) here is that it is midwives who instigated this change and individual midwives who decide which women are cared for on the pathway, using eligibility inclusion and exclusion criteria. This is a significant shift in professional authority, as traditionally the categorisation of women in terms of their perceived risk status was undertaken by obstetricians. Once women are being cared for on the pathway, they are categorised as being a midwife′s case, with no medical contact unless the midwife deems this necessary. As the pathway is used exclusively by midwives, the midwife controls access to the client and her clinical records. As one midwife explained:

I think it does sort of put a *little tag* [our emphasis] on that woman as a way of saying, ‘leave her alone’. Which I think some doctors respect and some don′t. (Focus group 1, Unit A midwives)

Interestingly, few doctors commented on how these initial eligibility decisions were made or on the shift in authority that accompanied them. Rather, as discussed in the next two themes, doctors’ concerns focused on altered professional boundaries and authority during intrapartum care and how they experienced these at the micro-level of service delivery.

The ‘normal labour pathway’ served an important function in legitimising midwifery authority. Midwives used it to emphasise their unique expertise in normal childbirth and to challenge the dominant biomedical discourse:

It takes a lot for the midwives to challenge the doctors because they are assumed to have the greater knowledge. But when it′s normal it′s our field and that′s where our strengths lie. (Focus group 5, Unit B midwives)

This exercising of midwifery authority was observed during fieldwork. For example, during one labour a doctor had entered the birth room uninvited and failed to introduce himself – both transgressions of the etiquette of midwife-led care. The midwife′s response is professionally polite but brief. While she does inform him that labour progress is slow (‘no change after 3 hours’), which could warrant a medical opinion, her clinical authority is evident: the woman′s progress is within normal boundaries and hence within the midwife′s jurisdiction:

Dr G Registrar

comes in to the

birth room: Is everything okay? [Does not introduce himself].

Midwife: Yes, she′s midwife-led care, 5 cms but no change after 3 hours. I′ve done an ARM, we′ll see what happens. (Field note 1 Unit A)

The pathway also potentially altered professional territories by both expanding and reducing the pool of women identified as ‘normal’, hence extending or constricting the midwives’ scope of practice. For example, as in field note 1 above, allowing more time for labour progress shifts the boundaries of normality, resulting in some women remaining in the midwife′s care rather than being transferred to medical care. In contrast, many midwives described the initial assessment criteria as too strict, so that women who would previously been viewed as low-risk were reclassified by midwives as a doctors′ case. Midwives questioned what the impact on these women might be (for example, they might now have electronic foetal monitoring during labour when this would not have happened previously). It was felt that a grey area had been created, where women were not low risk enough to meet the inclusion criteria for pathway care but were also not high risk. This problem was compounded when pathway eligibility determined place of birth; for example, a midwife-led birth centre or an obstetric unit. By shifting the parameters of normality the pathway thus had territorial implications for both midwives and doctors. Paradoxically, the increased status of midwifery work appeared to have come at the price of a reduced scope of practice.

### Shifting medical territory and jurisdiction

The doctors described increased tensions in their working relationships with midwives that they attributed to the pathway implementation, and expressed concerns about perceived shifts in professional authority and its implications, as in the following observation:

Dr G has very strong feelings about the pathway and protocols in general … He feels that the current problem with the pathway – but also I pick up, with childbirth in UK generally – is that doctors don′t see enough of the mothers, only called in to bail out the midwives. Because they don′t care for women throughout, don′t get sense of overall picture. He fears that there is a hidden agenda behind the pathway – that it will lead to centralisation of services, closure of smaller units. (Field note 1 Unit A)

The data indicated that many doctors were initially unaware of the significance of the pathway′s uni-professional focus, assuming that this was a midwifery initiative of little relevance to them. This may explain the apparent lack of lobbying to increase medical representation on the steering group.

Feelings of exclusion were a key feature of the doctors’ accounts, resulting primarily from the shift in occupational jurisdiction. Doctors no longer had automatic access to low-risk clients or their records of care. They expressed concerns that this limited knowledge could prove problematic if medical advice was required:

I think it′s better even if she′s midwifery-led care to be aware of what′s going on because I don′t like to work by crisis … The patients who are not on the pathway – we are briefed what′s going on, so we know because we are acting in control. [But] here′s somebody who is still a patient on labour ward – in the same setting that we are dealing with, only you don′t know anything about the patient until the patient runs into a problem. (Interview M, doctor Unit A)

Many of the doctors described how the pathway had resulted in midwives becoming ‘territorial’. There was frequent reference to boundaries being drawn on the basis of women being on the pathway:

Often, when we are doing ward rounds we′re told that one′s on the pathway, that one′s not on the pathway, you′re not going in there. I mean it′s not rigid but it is a bit territorial. (Interview J, doctor Unit B)

The doctors emphasised the value of team-working and how this had been compromised by the pathway:

I don′t believe that just no medical input is the best way because we work as a team. For me it is that ‘no intervention unless it is necessary’…. Too many Caesareans is not nice. It is not one thing, it′s the overall structure, which includes midwives, doctors, junior staff and the whole team approach that is important, and at the moment the pathway involves only the midwives. (Interview S, doctor Unit A)

The doctors also expressed concerns that they had no overview of the overall maternity unit workload, which they considered necessary to plan their working day. This overview also created a sense of ‘acting in control’, suggesting that even though they valued teamwork, it was teamwork with the doctor at the helm. The exclusion of the doctors was manifested in the use of the whiteboard. Previously used to provide an at-a-glance summary of all women admitted to the labour ward, with details of parity, gestation and other notable clinical features, for women on the pathway it now contained only the statement ‘midwife-led care’ (MLC):

If I come to the board, nobody′s going to tell me. I just read ‘MLC’. So all this says is that it′s midwife-led care. They won′t tell me anything more than that. But I know if there′s a problem, I′ve got to go in and it′s like you are basically called in to bail out. (Interview M, doctor Unit A)

Some midwives acknowledged that the shift in professional territories presented difficulties for the doctors; however the need to ‘keep the doctors out’ was generally accepted as inevitable in order for the midwives to establish the new order. The pathway was used as a boundary marker, with the phrase ‘on the normal care pathway’ being used to signify these altered territories:

They do their rounds on labour ward and you tell them ‘Oh, we are okay in here. She is on the normal care pathway and progressing fine’. (Focus group 2, Unit A midwives)

As doctors were not involved in decisions about whether women should commence or exit care on the pathway, they were highly reliant on the midwives’ clinical judgement. The doctors worried whether the pathway inclusion/exclusion criteria were always applied appropriately and whether midwives would rigidly stick to the pathway rather than using their clinical judgement, for example, in the case of slow progress in labour. Underpinning the doctors’ unease were differing – and indeed conflicting – professional understandings of normality, abnormality and risk, and how best to respond to the uncertainty and unpredictability of childbirth.

These dissonant approaches are evident in the following accounts. The doctor refers to ‘patients’ being ‘delivered’, emphasising both the dangerous nature of birth and the importance of medical knowledge. In contrast, the midwife expresses confidence in normal physiology and ‘women′s’ autonomy:

From what I understand, a normal care pathway means that this patient is presumed absolutely normal and will have absolutely normal labour, which I have a big reservation about because in labour, even if the patient had no problems before, you never know until the patient is delivered and the placenta is out … you see the problem with obstetrics is that some of them are very, very dicey and dangerous … Although the patient is OK at the moment then things can go pear-shaped any time. (Interview M, doctor Unit A)

They [doctors] just don′t have that belief in normal physiology. It′s sad to me, they just cannot believe that women will get on and do it themselves if you give them a chance to do it … They′ve got to be seen to be doing things. They get their hand in, rather than say ‘Hang on a minute, just step back. Let her be given a bit longer’. (Interview E, manager Unit A)

### Redefining the doctor′s role

The shift in territory resulted in changes to the doctors’ role. The doctors commented that it was now their technical skills rather than their interpersonal skills that were required, using phrases such as ‘bailing out’ and ‘coming in like the fire brigade’ to describe experiences that emphasised their instrumental function: ‘So we are placed in a much more technical position, and not so much a human position I think [interview J, doctor Unit B].

Although this emphasis on technical skill might be expected, given that obstetricians are the experts in abnormal childbirth and employed precisely because of this clinical expertise, in the view of the medical participants this aspect had been accentuated since the introduction of the pathway. Explaining why the lack of contact with low-risk women was problematic, doctors described their loss of the soft side of care:

There is a loss of that relationship [with women] and also the loss of being present with more normal deliveries. So I think that it′s a poorer experience for us…. If you want doctors to be holistic practitioners, then you should give them a chance – we have been pushed into this technical area. (Interview J, doctor Unit B)

The findings indicate that the ‘normal labour pathway’ was used by midwives to legitimise a midwifery model of care and redefine midwifery territory. As a result, medical territory and jurisdiction were altered and the doctor′s role was redefined. This in turn affected inter-professional relationships between midwives and doctors, with the clinical pathway acting at the interface of professional boundaries by signalling the borders of delegated authority.

## Discussion

The implementation of the ‘normal labour pathway’ led to changes in occupational jurisdiction and a shift in professional territories in Welsh maternity care, illuminating the differing professional perspectives of midwives and obstetricians and the contrasting underpinning models of childbirth that they held. It achieved these changes in three key ways. Firstly, it was used by midwives to delineate spheres of professional practice and the boundaries between them. Secondly, it identified the characteristics of midwife-led care; finally it determined at what point doctors should potentially re-enter the picture and become involved in women′s care. Even though the pathway did not fully involve doctors, they were part of its overall structure and it is therefore not surprising that it affected their roles and identity. The development of the pathway thus comprised key characteristics of a professional project that have been identified in the literature – the seeking of autonomy and the attempt to achieve occupational closure over an area of practice (Evans 2003, Witz 1990). For Witz (1992) a professional project is ‘a strategic course of collective action’ which acts to close off aspects of work to other groups and ‘employ[s] distinctive tactical means in pursuit of the strategic aim’ (p. 64). The pathway sought to achieve this closure by drawing a clear boundary between the sphere of normal labour (the expanded domain of midwives alone) and high-risk labour that required the medical and technical expertise of obstetricians in collaboration with midwives.

Unlike other clinical pathways aimed at enhancing inter-professional dialogue, the ‘normal labour pathway’ was never intended to dissolve professional boundaries. Rather than adopting a collaborative approach from the outset, with both professions discussing possible joint solutions to a shared problem, engagement with doctors was minimal. Indeed, the data suggest that doctors were frequently seen as part of the problem, not part of the solution. Limited engagement meant little opportunity to allay doctors’ fears about the safety of this new approach or garner their support. Moreover, this approach failed to acknowledge that medical participation would sometimes be clinically necessary. It is likely that this fundamental gulf in the social meaning of the pathway for all concerned led to the increased inter-professional tensions identified in this study. As Martin *et al*. (2009) suggest, ‘The sociological literature teaches us that professions tend to defend their jurisdictions fiercely, and respond to incursions by reasserting the legitimacy of existing boundaries′ (Martin *et al*. 2009: 1191).

Pathways are not ideologically neutral. Instead, they are socially and professionally constructed and draw on specific kinds of knowledge and belief systems (Berg 1997). Differing professional ideologies play out in both their creation and implementation. In the case of the ‘normal labour pathway’ it is the ideology of the midwifery profession, usually characterised as subordinate, which has been influential. Midwives employed the pathway to add legitimacy to their care and challenge the dominance of biomedical knowledge. As one midwife explained: ‘it puts a tag on the woman’, making midwifery work visible. By categorising women as being ‘on the pathway’ or ‘off the pathway’, the pathway has sharply re-drawn the frontiers of inter-professional territories, expanding midwifery territory whilst reducing medical territory. Paradoxically, the risk criteria used to redraw these professional boundaries resulted in an increase in midwifery power, but within the smaller jurisdiction of midwife-led care. Although medical surveillance is not permitted within this space the overall jurisdictional space of midwives has been decreased. As Scamell and Alaszewski (2012: 210) observe, there is ‘an ever closing window of normality’ created by the dominance of a risk discourse in UK maternity care. It is an irony that the pathway employed the language of risk to underpin its aim of supporting normal birth and, as a consequence, further shrank the pool of low-risk women. It may be that this outcome is a likely consequence of any midwife-led care and would certainly be worth further exploration.

This ethnographic study was conducted in two maternity units in Wales and cannot claim generalisability. As a major aim was to explore how the pathway was used and experienced by midwives, its key users, the sample of midwives, was considerably larger than the sample of doctors. This difference in sample size is acknowledged as a limitation in relation to our analysis of the pathway′s effects on inter-professional relationships. However, dissemination events held across Wales and attended by doctors and midwives indicated that the experiences of increased inter-professional tensions reported here were common, enhancing the study′s credibility.

It should also be noted that the study was completed in 2006, at a relatively early stage of pathway implementation when tensions would have been most keenly felt. A similar study conducted now would be likely to generate different findings. The ‘normal labour pathway’ remains in use throughout Wales and has been the subject of reviews in 2007 and 2012, with a revised ‘normal labour pathway’ being introduced in April 2013. The aims of the pathway, however, remain the same. The revisions relate mainly to the flow of the documentation (so that it better helps to tell the story of a woman′s labour) and the inclusion of substantive reference to NICE intrapartum care guidance, (National Collaborating Centre for Women and Children′s Health 2007) as the underpinning evidence base. There have also been changes in Welsh maternity policy over this period, with a *Strategic Vision for Maternity Services in Wales* published in 2011 (Welsh Government 2011). This strategy suggests a policy shift: the new focus is largely on public health, with no mention of enhancing the normal birth rate or of the ‘normal labour pathway’.

The ‘normal labour pathway’ has acted as a discursive tool to support a professional project led by midwives, whereby they can re-establish their professional territory and claim ownership of a distinctive body of knowledge in relation to normal birth. In doing so, it has disrupted the negotiated order with obstetrics. Whether this will lead to sustainable changes in inter-professional boundaries or whether each will ‘retreat towards the protected core’ (Larson, 1990: 45) remains to be seen.
